# KMT2A重排急性白血病异基因造血干细胞移植进展

**DOI:** 10.3760/cma.j.cn121090-20231026-00230

**Published:** 2024-05

**Authors:** 竞 刘, 晓军 黄

**Affiliations:** 北京大学人民医院，北京大学血液病研究所，国家血液系统疾病临床医学研究中心，造血干细胞移植北京市重点实验室，北京 100044 Peking University People's Hospital & Peking University Institute of Hematology, National Clinical Research Center for Hematologic Disease, Beijing Key Laboratory of Hematopoietic Stem Cell Transplantation, Beijing 100044, China

## Abstract

KMT2A重排急性白血病是具有独特生物学特性的一类白血病，其预后中等或不良。近年来，异基因造血干细胞移植（allo-HSCT）在KMT2A重排急性白血病中的应用日趋广泛。本文通过综述allo-HSCT治疗KMT2A重排急性白血病临床研究进展，分析allo-HSCT在儿童及成人KMT2A重排急性髓系白血病和急性淋巴细胞白血病中的疗效，归纳影响allo-HSCT预后的因素，并探讨可能改善allo-HSCT预后的方法。

## Abstract (en)

KMT2A（lysine methyltransferase 2A）-rearranged acute leukemia is a class of leukemia with unique biological characteristics with moderate or poor prognosis. In recent years, allogeneic hematopoietic stem cell transplantation（allo-HSCT）has been increasingly indicated for patients with KMT2A-rearranged acute leukemia. By reviewing the clinical studies of allo-HSCT in KMT2A-rearranged acute leukemia, the efficacy of allo-HSCT in children and adults with KMT2A-rearranged acute myeloid leukemia and acute lymphoblastic leukemia was assessed, the factors affecting the prognosis of allo-HSCT were summarized, and the methods that may improve the outcomes of allo-HSCT were explored.

KMT2A重排（KMT2A-r）急性白血病是具有独特生物学特性的一类白血病，其预后中等或不良。近年来，异基因造血干细胞移植（allo-HSCT）在KMT2A-r急性白血病中的应用日趋广泛。本文通过综述allo-HSCT治疗KMT2A-r急性白血病临床研究进展，分析allo-HSCT对于儿童及成人KMT2A-r急性髓系白血病（AML）和急性淋巴细胞白血病（ALL）的疗效，归纳allo-HSCT的预后影响因素，并探讨可能改善allo-HSCT预后的方法。

一、KMT2A基因

KMT2A基因也称作MLL（mixed lineage leukemia）基因，位于人类染色体的11q23.3区域，累及11q23染色体的KMT2A基因重排是急性白血病中可重现基因异常[1]–[3]。KMT2A基因事件分为平衡染色体互换易位和非平衡染色体异常，包括基因内重复、第11染色体上的缺失或倒位、KMT2A基因插入其他染色体中以及伙伴染色体物质插入到位于11q23.3的KMT2A基因中[4]–[5]。迄今为止已鉴定了超过135种KMT2A-r，其中7种最常见的是AFF1、MLLT3、MLLT1、MLLT10、AFDN、KMT2A-PTDs和ELL，占所有KMT2A-r的90％以上[4]–[5]。KMT2A-r在1岁以下婴儿ALL中发生率最高，在儿童和青年阶段发生率下降，直到55岁及以后略有增加[4]–[5]。2023年Meyer等[4]报道了国际多中心3401例KMT2A-r急性白血病特征，在KMT2A-r ALL中，最常见的3种KMT2A-r伙伴基因（AFF1、MLLT1和MLLT3）占所有ALL患者的87％，而在KMT2A-r AML中，最常见的6种KMT2A-r（MLLT3、MLLT1、MLLT10、AFDN、KMT2A-PTDs、ELL）占82％[4]–[5]。

二、KMT2A-r与急性白血病预后

KMT2A-r与急性白血病的发生和预后密切相关，最近版本的ELN和NCCN指南将AML伴t（9;11）（p21.3; q23.3）/MLLT3∷KMT2A和KMT2A-PTD划分为预后中等的细胞遗传和分子生物学组，而伴其他类型的t（v;11q23）/KMT2A-r则划分至预后不良，将ALL伴t（v;11q23）/KMT2A划分至预后不良[1]–[2]。不同KMT2A-r类型急性白血病的预后存在异质性。

早期文献主要研究了平衡11q23染色体重排AML的预后，在小样本病例研究中发现t（9;11）/KMT2A∷MLLT3 AML的总生存（OS）期（13个月）接近无11q23染色体异常的中等预后核型AML，而其余类型t（v;11q23）/KMT2A-r AML的OS显著差于无11q23染色体异常的中等预后核型AML，与预后不良核型AML接近，因此，将t（9;11）病例划分为预后中等组，而将其他t（v;11q23）归入预后不良细胞遗传学组[6]–[7]。近期更大病例数的研究证实了t（9;11）/KMT2A∷MLLT3 AML预后与无11q23染色体异常中等预后核型AML相近，并进一步对t（6;11）、t（10;11）、t（v;11）和非平衡11q23染色体异常AML进行了亚组分析，一致认为t（6;11）、t（10;11）重排AML预后不良，而其他不同类型KMT2A-r AML预后仍存在较大异质性[8]。例如t（11;19）（q23,p13）/KMT2A∷MLLT1的预后在不同研究中存在争议，Chen等[8]报道其预后不良，而Grimwade等[9]报道其预后中等。KMT2A-r ALL整体预后较差且存在异质性，最常见的t（4;11q23）/KMT2A∷AFF1在多项研究中显示出较其他类型更差的OS和无白血病生存（LFS）[10]–[14]。

儿童KMT2A-r急性白血病的预后同样存在异质性。婴儿（≤1岁）ALL中KMT2A-r发生率为75％～80％，长期LFS率约为50％。一项纳入619例儿童KMT2A-r ALL的研究报道的5年无事件生存（EFS）率在t（9;11）T-ALL、t（4;11）B-ALL和t（11;19）T-ALL中为41％～91％，存在较大的异质性[15]。而在一项纳入750余例KMT2A-r儿童AML的报道中，t（6;11）、t（10;11）和t（4;11）AML患者的5年EFS率仅为10％～40％[16]。

三、不同人群KMT2A-r急性白血病的allo-HSCT

1. 儿童KMT2A-r ALL：我中心Bai等[17]分析了37例伴KMT2A-r儿童B-ALL，其中19例患者在第1次完全缓解（CR1）时行allo-HSCT（以单倍体移植为主），allo-HSCT组、化疗组的4年OS率分别为（87.4±8.4）％、（52.2±12.4）％（*P*＝0.022），LFS率分别为（89.5±7.0）％、（25.9±10.7）％（*P*<0.001）。日本进行了3项研究，尝试通过对所有KMT2A-r ALL行allo-HSCT以改善预后，但结果显示仅具有高危因素的患者可能获益。MLL-10研究将KMT2A-r ALL患者根据是否有KMT2A-r、年龄和有无中枢神经系统白血病（CNSL），将KMT21-r ALL患儿分为中度风险（IR）和高危（HR）组，仅对HR组（年龄<180 d、CNSL、第8天泼尼松反应差）行HSCT，IR组患者的5年LFS率为94.4％，而HR患者为56.6％（*P*＝0.001），该研究还显示巩固化疗后的微小残留病（MRD）状态是影响LFS最重要的独立危险因素[18]。一项COG的研究报道<1岁KMT2A-r ALL在CR1期行allo-HSCT和化疗相比，5年LFS分别为48.8％和48.7％（*P*＝0.60）[19]。上述研究提示对所有KMT2A-r ALL患儿行allo-HSCT是不必要的。Interfant-99研究发现在具有高危因素的KMT2A-r ALL婴儿在CR1时接受allo-HSCT，4年LFS率高于化疗组（59.0％对22.2％，*P*＝0.01），而在其他KMT2A-r ALL婴儿，allo-HSCT组与化疗组4年LFS率分别为53.8％、60.9％（*P*＝0.09）[20]。Interfant-06研究中，移植适应证除了上述高危因素外，还增加了MRD≥0.01％，但其OS率较Inerfant-99无明显进步[21]。Attarbaschi等[15]回顾分析了629例伴KMT2A-r儿童ALL，其中107例在CR1时行allo-HSCT，但无论在总体人群、t（4;11）B-ALL亚组和KMT2A-r T-ALL中，allo-HSCT与化疗相比其预后差异无统计学意义。综上，具有高危因素的KMT2A-r婴儿ALL在CR1期行allo-HSCT可改善预后，而在其他人群CR1期allo-HSCT能否获益尚存争议。

2. 儿童KMT2A-r AML：Bai等[22]分析了我中心44例伴KMT2A-r儿童AML，所有患者在CR1时行allo-HSCT（以单倍体移植为主），3年OS率、EFS率、累积复发率（CIR）分别为（74.5±7.2）％、（64.1±8.0）％、（29.1±7.7）％。AML-BFM 98研究根据是否有可用同胞全相合供者将KMT2A-r AML儿童随机分为化疗组、allo-HSCT组，5年OS率分别为52％、94％（*P*＝0.01）[23]。一项对KMT2A-r AML儿童接受吉妥珠单抗后进行巩固allo-HSCT研究报告显示5年LFS率为72％，CIR为28％，明显优于化疗或移植前不加吉妥珠单抗的患者[24]。Romy等[25]在高危KMT2A亚型儿童AML进行的研究显示，CR1期行allo-HSCT可降低CIR，但对OS无影响，该研究中高危KMT2A亚型定义为6q27（KMT2A::AFDN）、4q21（KMT2A::AFF1）、10p12（KMT2A::MLLT10）、10p11.2（KMT2A::ABI1）、19p13.3（KMT2A::MLLT1）。综上，在高危儿童KMT2A-r AML患者中，CR1期行allo-HSCT可降低复发从而改善生存，单倍体移植可能获得和同胞全相合移植相似的生存，但鉴于不同类型KMT2A-r AML预后具有异质性，allo-HSCT在不同类型KMT2A-r AML中的作用需进一步研究。

3. 成人KMT2A-r AML：2014年我中心的前瞻性单臂队列研究（ChiCTR-ONC-12002739kjkl）纳入56例伴11q23异常成人急性白血病（<60岁），allo-HSCT后3年CIR为25.3％，4年OS率、LFS率分别为61.8％和56.3％，显著优于单纯化疗的29例同期对照病例，值得注意的是在该研究中有34例单倍体移植，多因素分析显示非CR1状态是影响移植后复发的独立危险因素[26]。Chen等[8]回顾性分析了24例伴11q23异常成人AML在CR1期行allo-HSCT（同胞全相合移植16例，无关供者移植4例，脐带血移植3例，单倍体移植1例），与仅化疗相比，移植组具有更长的OS期和LFS期（*P*<0.001），移植组的5年OS和LFS接近40％，而化疗组5年OS率、LFS率分别为13％、10％，移植在t（9;11）（p22;q23）/KMT2A-MLLT3 AML和其他t（v;11q23）AML中都表现出生存优势。2015年，欧洲血液与骨髓移植（EBMT）登记组[27]报道了159例11q23/MLL重排AML患者在CR1（138例）或CR2期行allo-HSCT的结果（同胞全相合移植79例，无关供者移植80例），2年OS率、LFS率、CIR、非复发死亡率（NRM）分别为（56±4）％、（51±4）％、（31±3）％、（17±4）％，该研究显示t（9;11）、t（11;19）AML的预后显著优于t（6;11）和t（10;11）AML，多因素分析显示t（6;11）、t（10;11）、年龄>40岁和CR2为OS的独立危险因素，而t（6;11）、t（10;11）、CR2和减低强度预处理是LFS的独立危险因素。2018年，日本造血干细胞移植协会（JSHCT）[28]报道了322例伴11q23异常成人AML的allo-HSCT数据，3年OS率、无复发生存（RFS）率、CIR和NRM分别为44％、38％、44％和18％，移植前非CR1状态是复发和生存的独立危险因素。该研究未发现不同KMT2A伙伴基因对移植预后的影响，但相比于复发率最低的t（11;17），t（6;11）AML有更高的复发率（*P*＝0.04），此外，该研究还发现移植前MRD阳性是影响移植后复发和生存的独立危险因素。综上，上述研究支持在CR1期行allo-HSCT可减少成人KMT2A-r AML复发，改善其长期生存，且有可能达到治愈，但其长期生存率仍较低，在具备高危因素的亚组中可能仍无法克服其较差的预后。

4. 成人KMT2A-r ALL：ECOG2993队列[29]2013年报道了85例t（4;11）重排ALL的数据，其中31例在CR1期接受了allo-HSCT（15例同胞全相合和16例非血缘供者），移植组5年OS率分别为56％和66％，显著高于未移植患者，但多因素分析并未发现移植的独立预后意义。EBMT白血病工作组2020年报道了151例t（4;11）重排前体B-ALL在CR1期行allo-HSCT（同胞全相合48例，无关供者103例），2年OS率、LFS率、CIR、NRM分别为60％、51％、30％、20％，与正常核型前体B-ALL相比差异无统计学意义，复发是导致移植后死亡最主要的原因，该研究还显示移植前MRD阴性是影响LFS、OS、CIR和NRM的有利因素[30]。除t（4;11）外的KMT2A-r在ALL中较为少见，其allo-HSCT数据也较少。2021年MD Anderson癌症中心分析了50例伴KMT2A-r ALL患者的治疗数据，KMT2A-r ALL总体预后较差，5年OS率仅18％，t（4;11）与其他KMT2A-r之间差异无统计学意义（*P*＝0.87），其中18例患者在CR1进行了allo-HSCT，移植患者的5年OS率优于非移植患者（32％对11％，*P*＝0.01）[31]。综上，成人KMT2A-r ALL在CR1期allo-HSCT有助于降低复发，改善生存，但其长期预后在不同报道中差异较大（5年OS率为32％～67％）。

四、影响KMT2A-r急性白血病CR期行allo-HSCT预后的因素

1. KMT2A-r伙伴基因：尽管目前仍有争议，但一些研究显示KMT2A-r的伙伴基因可能影响急性白血病（尤其是AML）患者的移植预后。EBMT的数据显示不同11q23/MLL重排成人AML其移植预后不同，t（9;11）和t（11;19）AML的生存显著优于t（10;11）和t（6;11）AML患者［2年OS率分别为（64±6）％、（73±10）％、（40±13）％、（24±11）％，*P*<0.001］，后两者较差的OS主要是由于移植后2年CIR仍高达50％[27]。然而JSHCT在相似病例数的研究中却未发现不同KMT2A-r伙伴基因对AML移植预后有显著影响，但相比于复发率最低的t（11;17），t（6;11）AML有更高的CIR（*P*＝0.04）[28]。目前为止最大病例数的研究来自国际血液与骨髓移植研究中心（CIBMTR）数据库，426例KMT2A AML患者在CR1期行allo-HSCT，未发现不同KMT2A伙伴基因对OS、LFS、CIR和NRM的影响[32]。在KMT2A-r成人ALL中，最常见的t（4;11）ALL与其他类型预后未见显著差异。

2. MRD：MRD是影响allo-HSCT后白血病复发重要的预后因素，急性白血病患者在治疗期间的随访和基于个体MRD监测的治疗调整对预后有非常强的影响，通过实时定量PCR（RT-qPCR）检测患者特异性的KMT2A融合序列可作为MRD标记[33]。

本中心开展了系列研究分析KMT2A融合基因和KMT2A-PTD对allo-HSCT预后的影响。Liu等[34]–[35]采用RT-qPCR方法动态检测在CR状态下行allo-HSCT的急性白血病患者移植前后KMT2A融合基因水平，发现移植前KMT2A水平与移植后KMT2A水平相关（*P*<0.001），在移植前KMT2A低水平组（>0～<0.1％）、中水平组（0.1％～<1％）和高水平组（≥1％）中，allo-HSCT后KMT2A转阴率分别为96.6％、92.9％和68.8％（*P*＝0.027）。移植前KMT2A阴性组、低水平组、中水平组和高水平组在移植后KMT2A阳性率分别为7.7％、35.7％、38.5％、45.5％（*P*<0.001）。移植前KMT2A≥0.1％组allo-HSCT后4年CIR高达53.7％，高于KMT2A阴性组（15.1％）和KMT2A<0.1％组（31.2％）（*P*<0.001），4年OS率和LFS率也差于KMT2A阴性和低水平组。Liu等[35]还报道移植后监测KMT2A融合基因水平可预测复发与生存，KMT2A阳性（>10^−4^）患者的3年CIR显著高于KMT2A持续阴性的患者（93.5％对12.5％，*P*<0.001），而3年OS率和RFS率显著差于KMT2A持续阴性患者（12.5％对77.8％，0％对72.2％，*P*<0.001）。Kong等[36]报道allo-HSCT后KMT2A-PTD≥1％的AML患者3年CIR高于KMT2A-PTD<1％的患者［（75±15.3）％对0％，*P*<0.001］，3年OS率、LFS率均差于KMT2A-PTD<1％的患者［（25.0±15.3）％对（80.7±6.6）％，*P*<0.001；（25.0±15.3）％对（80.7±6.6）％，*P*<0.001］。

几项国外研究同样显示移植前KMT2A水平是影响预后的独立危险因素。Konuma等[28]报道移植前KMT2A阳性AML后复发和生存均差于KMT2A阴性患者，MRD阳性组、阴性组3年CIR分别为46％、26％（*P*＝0.02），3年OS率分别为39％、64％（*P*＝0.02）。EBMT白血病工作组报道t（4;11）重排前体B-ALL移植前MRD阴性是影响LFS、OS、CIR和NRM的最强有利因素，移植前MRD阴性t（4;11）患者可获得与MRD阴性正常核型患者相似的预后，但MRD阳性t（4;11）患者的LFS率差于MRD阳性正常核型患者（27％对50％，*P*＝0.02）[30]。

3. 治疗相关和继发急性白血病：KMT2A-r比较常见于治疗相关急性白血病（t-AL）和继发急性白血病（s-AL），尤其是成人t-AML/s-AML[6],[8],[37]。尽管t-AL/s-AL通常被认为是不良预后因素，但目前研究显示其在KMT2A-r急性白血病中似乎预后意义较弱。Scoch等[6]研究显示KMT2A-r在转化的AML（t-AML）中的发生率显著高于原发AML（9.4％对2.6％，*P*<0.001），且t-AML的中位OS期差于原发AML（2.5个月对10个月，*P*＝0.0143）。然而，Menghrajani等[37]和Chen等[8]在不同的研究中发现KMT2A-r t-AML的生存与原发AML并无差别，两个研究均发现在t-AML中9p22基因伙伴占比更高，而这种基因亚型通常被认为是KMT2A-r AML中生存最好的亚型。Issa等[38]研究显示t-AML在KMT2A-r AML中是独立的不良预后因素，但当把allo-HSCT纳入多因素分析后，t-AML不再具有独立预后意义。同样，在多数移植治疗KMT2A-r急性白血病的研究中也未发现t-AML与原发AML之间的预后有差异[15],[24],[27]–[28],[30]–[32]。综上，s-AL和t-AL在KMT2A-r急性白血病中是较弱的预后因素。

4. 其他因素：文献报道其他影响KMT2A-r急性白血病移植预后的因素还有年龄（>40岁）、CR2、供者类型、预处理强度、伴随染色体异常等，但由于人群、所设阈值等差异，在不同研究之间未达成一致的结论[26]–[28],[32],[34],[36]–[37]。

综上，影响KMT2A-r急性白血病allo-HSCT预后的危险因素尚未被充分阐明，目前数据显示移植前和移植后MRD是影响KMT2A-r急性白血病移植预后的独立危险因素，KMT2A伙伴基因类型的预后意义目前存在争议。

五、改善KMT2A-r急性白血病allo-HSCT预后的策略

迄今为止，KMT2A-r急性白血病的长期预后仍较差，亟需探索新治疗手段。可能有效的治疗手段包括：增强allo-HSCT后移植物抗白血病（GVL）效应，加强预处理方案或将新型治疗方法（免疫治疗、DOT1L1抑制剂、Menin抑制剂、HDAC抑制剂等）用于难治/复发患者的移植前治疗或移植后复发防治。

1. MRD指导的抢先干预免疫治疗：MRD指导的抢先干预免疫治疗是防治allo-HSCT后复发的重要手段，研究显示，MRD指导下的抢先化疗联合供者淋巴细胞输注（DLI）和α干扰素治疗可有效减少急性白血病复发，改善长期生存[39]–[43]。本中心回顾性分析了36例KMT2A融合基因阳性急性白血病患者allo-HSCT后的预后，其中有27例在KMT2A阳性时进行了免疫治疗抢先干预，其中大部分（25/27）是AML，其中21例患者接受了α干扰素治疗，19例患者进行了化疗联合DLI（其中13例为α干扰素治疗后KMT2A阳性），结果显示α干扰素治疗后有33.3％的患者KMT2A转阴，但大部分患者再次转阳，2例（9.5％）患者获得无病生存，化疗联合DLI后有47.3％的患者KMT2A转阴，4例（21.1％）患者获得无病生存。接受、未接受抢先干预免疫治疗患者的中位OS期分别为717 d、368 d（*P*＝0.352），RFS期分别为359 d、297 d（*P*＝0.638）[34]。该研究显示抢先干预免疫治疗可能有助于KMT2A转阴，但其长期疗效仍不满意，因上述研究为回顾性资料且例数较少，抢先干预免疫治疗在KMT2A-r急性白血病中的疗效尚待通过前瞻性设计队列继续研究，其机制也有待进一步阐明。

2. 移植后的维持治疗：移植后维持治疗有助于清除白血病残留，是减低移植后复发的重要策略。虽然目前还没有被批准用于移植后的维持治疗，但有许多相关研究和正在进行的临床试验，例如去甲基化药物、新型分子靶向药物、免疫调节治疗和细胞治疗等[44]–[46]。Wang等[44]研究显示对进展期急性白血病患者，在allo-HSCT后60 d行预防性DLI可显著降低复发率，而对NRM无影响。一项Ⅱ期前瞻性随机对照研究显示粒细胞集落刺激因子（G-CSF）联合小剂量地西他滨用于allo-HSCT后维持治疗，将高危AML患者的移植后2年CIR降至15％，而未接受地西他滨维持治疗的患者为38.3％[45]。目前我中心对移植前未达CR状态的KMT2A-r急性白血病行预防性DLI，而其他KMT2A-r急性白血病患者的维持治疗还需要通过前瞻性随机对照试验探究。

3. 新型药物：随着对KMT2A-r急性白血病发病机制的研究，越来越多的证据表明，大量的表观遗传调控因子直接或间接参与了KMT2A-r急性白血病的发生与进展，表观遗传修饰可以通过药物靶向相关的表观遗传调节因子来逆转，此外，许多表观遗传酶也参与了DNA损伤反应（DDR），可能是潜在的治疗靶点[47]。一些表观遗传调控因子，如DOT1L、KDM4C和PRMT1，被证明通过KMT2A-r被招募到KMT2A靶基因位点参与白血病的发生[48]–[50]。相应的小分子抑制剂（如DOT1L抑制剂、KDM抑制剂、MLL-Menin抑制剂和PRMT抑制剂等）正在研发中，以逆转KMT2A靶基因位点的表观遗传状态，从而抑制其表达。尽管在体外有抑制KMT2A白血病细胞的作用，但许多小分子药物（如HDAC抑制剂）在体内单药应用时并未取得满意的疗效[51]。Menin是KMT2A靶基因激活和随后的白血病转化中的必需因子，因此成为KMT2A白血病的潜在药物靶点[48],[52]。Menin抑制剂Ⅰ期临床试验治疗难治复发KMT2A-r或NPM1突变急性白血病显示出较低的毒性和约30％的CR率，多种Menin抑制剂的临床研究正在进行中[47],[52]。此外，一项对KMT2A-r AML儿童接受吉妥珠单抗后进行巩固移植的研究显示，移植后5年EFS率为72％，CIR为28％，明显优于化疗和移植前不加吉妥珠单抗的患者[24]。

4. 免疫治疗：包括CAR-T细胞、Blinatumomab等免疫治疗方法在B-ALL已取得较好的疗效。虽然KMT2A-r ALL患者已被允许使用免疫治疗，但相关研究纳入的KMT2A-r ALL病例数有限，而且KMT2A-r ALL在CAR-T和Blinatumomab治疗中表现出一些独特的特征（如白血病细胞谱系的转换），其疗效有待研究[53]–[55]。复发难治、持续MRD阳性等KMT2A-r急性白血病患者allo-HSCT的预后仍差，在此类患者中结合免疫治疗有望改善预后。

六、总结

总之，allo-HSCT是治疗成人KMT2A-r急性白血病和具备高危因素儿童KMT2A-r急性白血病的有效方法。迄今为止，KMT2A-r急性白血病的长期预后仍不佳，影响其allo-HSCT预后的危险因素尚未完全阐明，移植前后监测MRD可能有助于预测allo-HSCT后急性白血病复发，指导分层治疗。还需探索新的治疗手段，以改善allo-HSCT预后。
